# Mutations in bassoon in individuals with familial and sporadic progressive supranuclear palsy-like syndrome

**DOI:** 10.1038/s41598-018-19198-0

**Published:** 2018-01-16

**Authors:** Ichiro Yabe, Hiroaki Yaguchi, Yasutaka Kato, Yasuo Miki, Hidehisa Takahashi, Satoshi Tanikawa, Shinichi Shirai, Ikuko Takahashi, Mari Kimura, Yuka Hama, Masaaki Matsushima, Shinsuke Fujioka, Takahiro Kano, Masashi Watanabe, Shin Nakagawa, Yasuyuki Kunieda, Yoshio Ikeda, Masato Hasegawa, Hiroshi Nishihara, Toshihisa Ohtsuka, Shinya Tanaka, Yoshio Tsuboi, Shigetsugu Hatakeyama, Koichi Wakabayashi, Hidenao Sasaki

**Affiliations:** 1 Department of Neurology, Faculty of Medicine and Graduate School of Medicine, Hokkaido University, Sapporo, Japan; 2 Department of Cancer Pathology, Faculty of Medicine and Graduate School of Medicine, Hokkaido University, Sapporo, Japan; 3 Department of Neuropathology, Hirosaki University Graduate School of Medicine, Hirosaki, Japan; 4 Department of Biochemistry, Faculty of Medicine and Graduate School of Medicine, Hokkaido University, Sapporo, Japan; 5 Department of Neurology, Fukuoka University School of Medicine, Fukuoka, Japan; 6 Department of Psychiatry, Faculty of Medicine and Graduate School of Medicine, Hokkaido University, Sapporo, Japan; 7 Wakkanai City Hospital, Wakkanai, Japan; 8 Department of Neurology, Gunma University Graduate School of Medicine, Maebashi, Japan; 9 Department of Dementia and Higher Brain Function, Tokyo Metropolitan Institute of Medical Science, Tokyo, Japan; 10 Department of Biochemistry, Faculty of Medicine/Graduate School of Medicine, University of Yamanashi, Chuo, Japan; 11 Laboratory of Oncology, Hokuto Hospital, Obihiro, Japan; 12 Division of Clinical Cancer Genomics, Cancer Center, Keio University School of Medicine, Tokyo, Japan; 13 Global Station for Soft Matter, Global Institution for Collaborative Research and Education, Hokkaido University, Sapporo, Japan

## Abstract

Clinical diagnosis of progressive supranuclear palsy (PSP) is sometimes difficult because various phenotypes have been identified. Here, we report a mutation in the bassoon (*BSN*) gene in a family with PSP-like syndrome. Their clinical features resembled not only those of PSP patients but also those of individuals with multiple system atrophy and Alzheimer’s disease. The neuropathological findings showed a novel three + four repeat tauopathy with pallido-luysio-nigral degeneration and hippocampal sclerosis. Whole-exome analysis of this family identified a novel missense mutation in *BSN*. Within the pedigree, the detected *BSN* mutation was found only in affected individuals. Further genetic analyses were conducted in probands from four other pedigrees with PSP-like syndrome and in 41 sporadic cases. Three missense mutations in *BSN* that are very rarely listed in databases of healthy subjects were found in four sporadic cases. Western blot analysis of tau following the overexpression of wild-type or mutated BSN revealed the possibility that wild-type BSN reduced tau accumulation, while mutated BSN lost this function. An association between *BSN* and neurological diseases has not been previously reported. Our results revealed that the neurodegenerative disorder associated with the original proband’s pedigree is a novel tauopathy, differing from known dementia and parkinsonism syndromes, including PSP.

## Introduction

Progressive supranuclear palsy (PSP) is a clinical syndrome comprising supranuclear palsy, postural instability, and cognitive decline. Neuropathologically, PSP is defined by neuronal loss in the basal ganglia and brainstem, with widespread occurrence of neurofibrillary tangles (NFTs)^1^. Recently, new insights have emphasized that the pathological events and processes leading to the accumulation of phosphorylated tau protein in the brain are best conceptualized as dynamic processes that can develop at different rates, resulting in different clinical phenomena. Moreover, for patients in whom the diagnosis is unclear, clinicians must continue to accurately describe the clinical situation in each individual instead of labelling them with inaccurate diagnostic categories, e.g., atypical parkinsonism or PSP mimics^1^. For example, there are patients with an atypical four-repeat tauopathy who do not satisfy the pathological diagnostic criteria for corticobasal degeneration (CBD) or PSP, despite the presence of neurodegeneration with tau-positive neuronal and glial cytoplasmic inclusions^2,3^. Although the disease entity has not been established, this type of atypical four-repeat tauopathy may be considered a differential diagnosis for corticobasal syndrome (CBS)^2^. With recent advances in genetics, new atypical parkinsonian conditions are emerging that share some clinical features with the classical phenotypes of PSP, CBD, and multiple system atrophy (MSA) and have therefore been described as PSP, CBD, or MSA ‘look-alikes’^4^. Genetic conditions can be diagnosed *in vivo* through genetic testing and may have different prognoses and be potentially treatable^4^.

PSP is usually sporadic, but a few pedigrees with familial clustering of PSP-like phenotypes have been described^5^. Mutations in *MAPT*, *C9ORF72*, *TARDBP*, *VCP*, and *CHMP2B* have been identified^6–8^. *DCTN1* mutations should be screened for in patients showing clinical PSP-like phenotypes and behavioural variants of frontotemporal dementia (FTD)^9^. FTD with parkinsonism-17 is also caused by mutations in the gene that encodes progranulin^10^.

In this report, we present a novel pedigree that displays a PSP-like syndrome and harbours a mutation in bassoon (*BSN*), and we present our analysis of *BSN* mutations in four hereditary and 41 sporadic cases with PSP-like syndrome.

## Results

### Identification of a Japanese family with PSP-like syndrome

**Figure 1:**
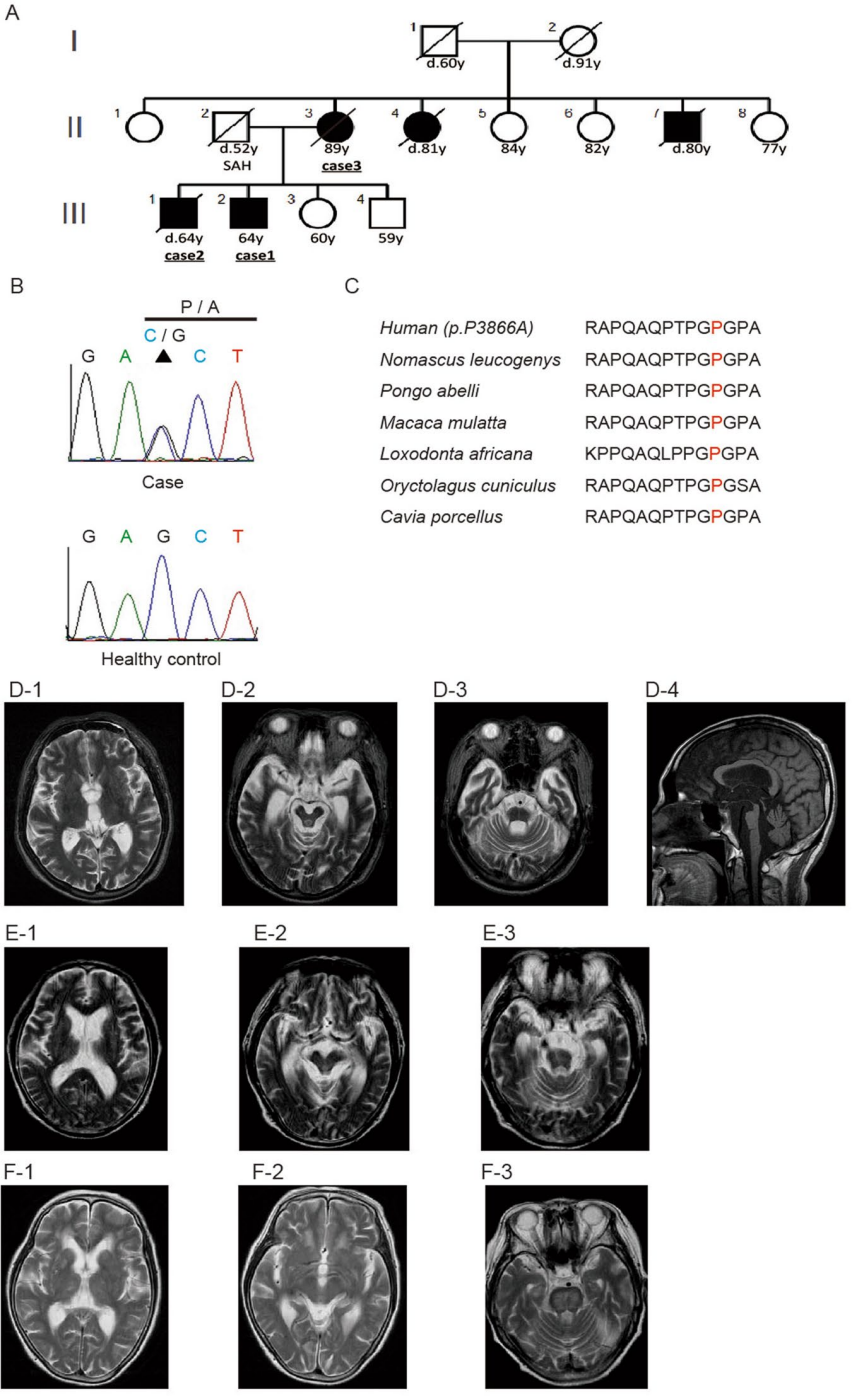
Sequence chromatograms of the c.11596C > G (p.Pro3866Ala) mutation in BSN and MRI findings. (**A**)Solid symbols indicate affected members; open symbols indicate unaffected individuals. (**B**) Chromatograms of a coding exon of the *BSN* gene. The c.11596C > G (p.Pro3866Ala) mutation is indicated by the arrowhead, and the corresponding normal sequence is shown below. (**C**) Evolutionarily conserved domains of the BSN p.P3866A mutation. (**D**) Sagittal T2WI and T1WI of the brain of individual III-2 (Case 1). This MRI analysis showed severe atrophy of the bilateral hippocampus, mesencephalic tegmentum, cerebellum, and brainstem. (**E**) T2WI of the brain of individual III-1 (Case 2). This MRI analysis showed severe atrophy of the bilateral hippocampus, mesencephalic tegmentum, cerebellum, and brainstem. (**F**) T2WI of the brain of individual II-3 (Case 3). This MRI analysis showed mild atrophy of the bilateral hippocampus, mesencephalic tegmentum, cerebellum, and brainstem compared with Cases 1 and 2.

Here, we describe a Japanese family with PSP-like syndrome. The family pedigree is shown in Fig. 1A. Three patients exhibited cognitive decline and postural instability. All of these patients fulfilled the National Institute of Neurological Disorders and Stroke (NINDS)-PSP diagnostic criteria^11^. Moreover, all affected individuals were classified as having PSP-FTD^12^. A summary of the clinical features of the patients is shown in Table 1.

**Table 1:** Clinical features of the affected family members.

	Case 1 (III-2)	Case 2 (III-1)	Case 3 (II-3)
Onset age (years)	43	52	83
Primary symptom	Dementia	Dementia	Dementia and anxiety
Disease duration (years)	18	12	9
Duration (years) from onset to gait disturbance	9	11	3
Rigidity of the neck	+++	+++	+
Postural instability	+++	+++	++
Supranuclear palsy	+	+	±
Atrophy of the temporal lobe	+++	+++	+
Atrophy of the brain stem	+++	+++	±
Hot cross bun sign	+	+	±
^123^I-IMP	Decrease in the temporal lobe and brainstem	Decrease in the temporal lobe and brainstem	Decrease in the temporal lobe and brainstem
MIBG	Normal	Normal	Normal

**Case 1:** After graduating from university, the patient (III-2) worked in a city hall. The onset of neurological symptoms consisted of memory disturbance at 43 years of age. Forgetfulness was first observed at ~43 years of age, then gradually worsened, and mild postural instability with a tendency to fall developed. The patient did not suffer from encephalitis. He was diagnosed with early-onset dementia by a local neuropsychiatrist, and progression of his symptoms was observed. At ~52 years of age, his tendency to fall as a result of postural instability and bradykinesia became severe. At 53 years of age, we performed a neurological examination of the patient for the first time, revealing severe cognitive decline (Mini-Mental State Examination 12/30), supranuclear palsy, severe neck rigidity, severe postural instability, and a grasping reflex. He could not walk by himself; however, severe asymmetric parkinsonian signs, alien limb syndrome, cortical sensory deficits and cerebellar ataxia were absent. T2- (T2WI) and T1-weighted imaging (T1WI) of the brain showed severe atrophy of the bilateral hippocampus, mesencephalic tegmentum, cerebellum, and brainstem (Fig. 1D). In particular, the ‘hot cross bun sign’ was detected in the brainstem (Fig. 1D). Single-photon emission computed tomography (SPECT) with ^123^I-IMP revealed hypoperfusion in the bilateral temporal lobes and cerebellum. Reduced cardiac uptake of ^123^I-metaiodobenzylguanidine (MIBG) was not detected. In the cerebrospinal fluid, the cell count was normal, and the protein level (102 mg/dL) was increased; however, the level of phosphorylated tau protein was not elevated. Treatment with L-DOPA did not improve his neurological symptoms. His gait and cognitive function deteriorated gradually. He was admitted to a care support hospital because of his disabilities and was bedridden at 58 years of age. Sleep apnoea and dysphagia were noted, and they subsequently intensified. Bi-level positive airway pressure was used, and gastrostomy was performed. A tracheotomy was then performed. When the patient was 64 years old, a neurological examination revealed decerebrate posturing, vocal cord paralysis, central-type apnoea, and severe neck rigidity.

**Figure 2:**
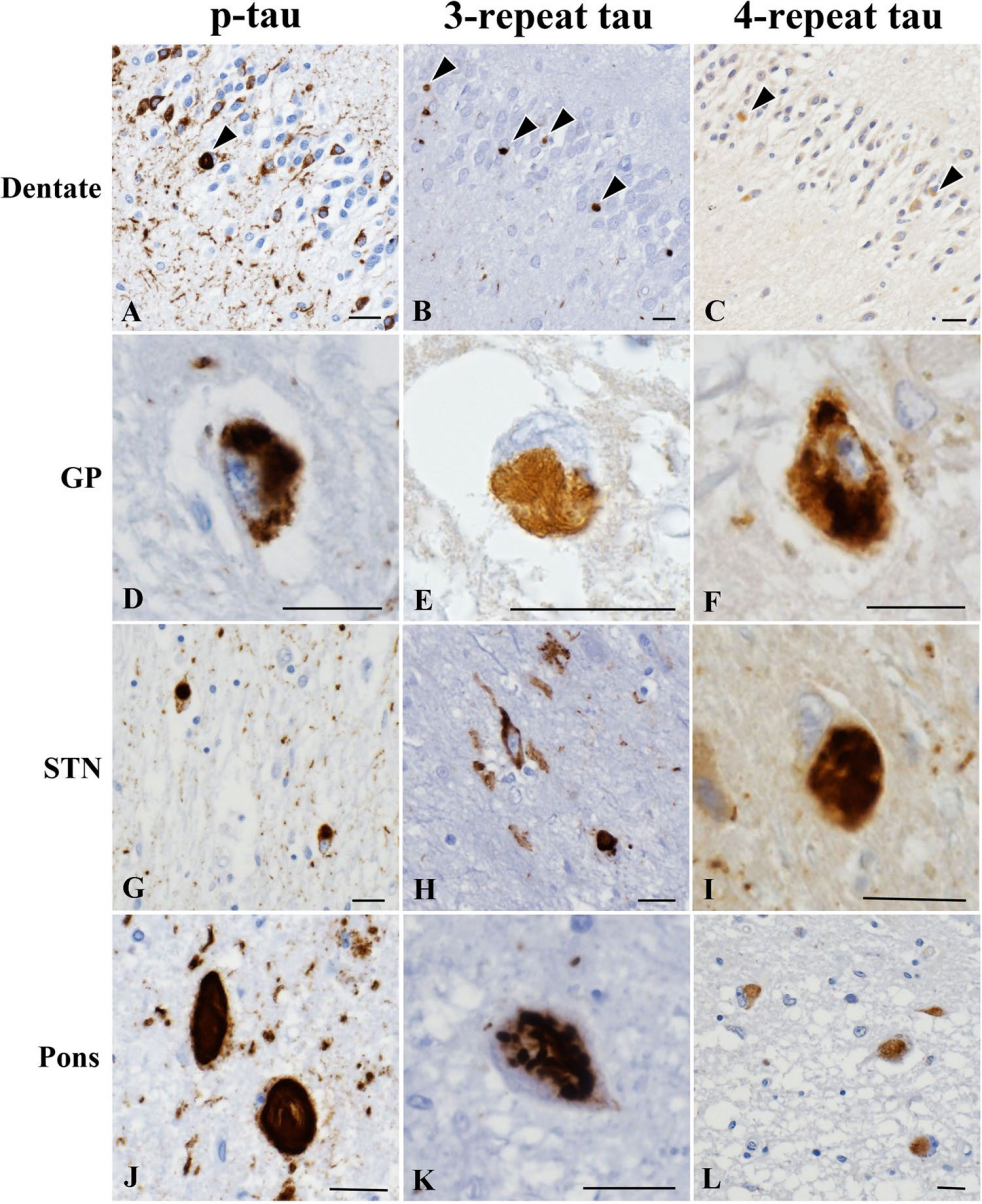
Pathological findings for individual III-1 (Case 2). Neurofibrillary tangles in the dentate gyrus (**A**–**C**, arrowheads), globus pallidus (**D**–**F**), subthalamic nucleus (**G**–**I**), and pontine tegmentum (**J**–**L**) are immunopositive for phosphorylated tau (**A**,**D**,**G**, and **J**), three-repeat tau (**B**,**E**,**H**, and **K**), and four-repeat tau (**C**,**F**,**I**, and **L**). Bars = 20 μm.

**Case 2:** The patient (III-1) was the older brother of Case 1 (III-2). His clinical course strongly resembled that of his brother. After graduating from university, he worked as a high school teacher. The onset of neurological symptoms consisted of cognitive decline at 52 years of age. He also exhibited progressive memory disturbance and mild postural instability with a tendency to fall. He did not suffer from encephalitis. When the patient was 57 years old, his cognitive decline worsened, causing him difficulties in carrying out daily activities, and the patient was diagnosed with Alzheimer’s disease by a psychologist. At ~63 years of age, his tendency to fall as a result of postural instability and bradykinesia became severe. At 64 years of age, we performed our first neurological examination of Case 2, revealing severe cognitive decline, supranuclear palsy, severe neck rigidity, severe postural instability, a grasping reflex, and apraxia of lid opening. He could not walk by himself owing to severe postural instability. However, severe asymmetric parkinsonian signs, alien limb syndrome, cortical sensory deficits and cerebellar ataxia were not observed. The findings from magnetic resonance imaging (MRI) (Fig. 1E), SPECT, and MIBG were similar to those of Case 1. An autopsy was implemented after Case 2 died of pneumonia at the age of 64 years. His neuropathological findings are shown in Fig. 2. His brain weighed 1,160 g. Gross examination revealed marked atrophy of the hippocampus and severe depigmentation of the bilateral substantia nigra. The pons was also atrophic. Histopathological examination revealed complete loss of hippocampal neurons. The frontal and temporal neocortices showed superficial spongiosis with a few ballooned neurons. The most marked changes in subcortical regions were observed in the substantia nigra and subthalamic nucleus. Severe neuronal loss with gliosis was found in these areas. Mild to moderate loss of neurons was also observed in the external segment of the globus pallidus, medial thalamic nuclei, brainstem tegmentum, and pontine base. Severe atrophy of the pons was detected, which appeared to be the underlying cause of the hot cross bun sign observed by MRI. The cerebellar dentate nuclei showed grumose degeneration. Gallyas-Braak staining and AT8 immunostaining revealed many NFTs in the hippocampus, globus pallidus, subthalamic nucleus, substantia nigra, and pontine tegmentum. A few NFTs were also observed in the frontal and temporal neocortices, thalamus, pontine base, and dentate nucleus. These NFTs were immunostained with both three-repeat and four-repeat tau antibodies (Fig. 2). Double-labelling immunofluorescence analysis revealed the co-localization of three-repeat tau and four-repeat tau in NFTs in the dentate gyrus (arrowheads) (see Supplementary Fig. S1).

Although a small number of coiled bodies were distributed in the subcortical nuclei, no other glial tau pathology (tufted astrocytes and astrocytic plaques) was present. Additionally, no amyloid deposits; Lewy bodies; or TDP-43−, FUS−, or polyglutamine-positive inclusions were observed. BSN expression in this patient was not reduced compared with that of age-matched normal controls (n = 5). Finally, his histopathological findings did not correspond to those of previously reported PSP or PSP-like syndrome patients. Furthermore, Western blot analysis of a freshly frozen sample of the frontal cortex revealed phosphorylated tau triplet bands (60, 64, and 68 kDa) (arrow) similar to those observed in patients with Alzheimer’s disease (see Supplementary Fig. S2) and did not reveal a decrease in BSN expression (see Supplementary Fig. S3). Next, to evaluate accumulation of tau as a result of the *BSN* mutation, we tested a normal brain and a patient’s brain for the *BSN* mutation. The accumulation of tau in the patient’s brain compared with the control brain was very clearly seen (see Supplementary Fig. S4).

**Case 3:** The patient (II-3) was the mother of Cases 1 (III-2) and 2 (III-1). After receiving compulsory education, she married and became a housewife. Her clinical course resembled those of Cases 1 and 2; however, the onset of her clinical symptoms was delayed. Her neurological symptoms consisted of memory disturbance at 80 years of age, progressive cognitive decline, and mild postural instability with a tendency to fall. She did not suffer from encephalitis. At ~83 years of age, she tended to fall as a result of postural instability and bradykinesia. At 89 years of age, we performed a neurological examination of Case 3 for the first time, revealing severe cognitive decline, supranuclear palsy, severe neck rigidity, severe postural instability, and a grasping reflex. She could not walk by herself owing to severe postural instability. However, severe asymmetric parkinsonian signs, alien limb syndrome, cortical sensory deficits and cerebellar ataxia were not observed. T2WI and T1WI of the brain showed milder atrophy of the bilateral hippocampus, mesencephalic tegmentum, cerebellum, and brainstem than in Cases 1 and 2. Furthermore, in this case, although the typical hot cross bun sign was not observed, abnormal high-intensity areas were observed upon T2WI of the pons (Fig. 1F). The patient’s SPECT and MIBG findings were similar to those of Cases 1 and 2. At the age of 90 years, she died of pneumonia; however, an autopsy was not performed.

One of the authors (IY or SS) examined II-5, II-6, III-3, and III-4, and no positive neurological findings were obtained.

### Identification of the point mutation c.11596C>G, p.Pro3866Ala in *BSN* in the Japanese family with PSP-like syndrome

Fifty-two select genes (Table 2) and whole mitochondrial DNA, which are the most common causes of Alzheimer’s disease, parkinsonism, mitochondrial diseases, FTD, PSP, and spinocerebellar ataxias, were analysed, but no mutations were identified^4,13^. Although linkage to 1q31.1 was reported in a large Spanish family with typical autosomal dominant PSP^14^, the family in our study did not show such linkage (LOD score < 0). Furthermore, we confirmed that each tau exon and splice region was adequately covered along with the selected genes, as the disorder within this family was considered a novel tauopathy. In addition, the LOD score of *PARK2*, *UCHL1*, and *ATXN3* was 1.4769, which was the maximum LOD score observed in this family (Table 2). Therefore, we screened these genes for copy number variations (CNVs), and no duplications or deletions of *MAPT*, *PARK2*, *UCHL1*, and *ATXN3* were detected through CNV and linkage analyses (Table 2).

**Table 2:** Fifty-two candidate genes and the LOD score of each in the pedigree.

No	Gene	Associated disease	Official name	Chromosome	Start*	End*	LOD score**
1	*APP*	Alzheimer’s disease	APP	21	27,252,861	27,543,446	Less than 0
2	*PSEN1*	Alzheimer’s disease	PSEN1	14	73,603,143	73,690,399	Less than 0
3	*PSEN2*	Alzheimer’s disease	PSEN2	1	227,057,885	227,083,804	Less than 0
4	*MAPT*	Frontotemporal lobar degeneration	MAPT	17	43,971,702	44,105,700	Less than 0
5	*C9ORF72*	Frontotemporal lobar degeneration	C9ORF72	9	27,546,543	27,573,864	Less than 0
6	*TARDBP*	Frontotemporal lobar degeneration	TARDBP	1	11,072,462	11,085,549	Less than 0
7	*FUS*	Frontotemporal lobar degeneration	FUS	16	31,191,431	31,206,192	Less than 0
8	*VCP*	Frontotemporal lobar degeneration	VCP	9	35,056,065	35,072,739	Less than 0
9	*GRN*	Frontotemporal lobar degeneration	GRN	17	42,422,491	42,430,474	Less than 0
10	*CHMP2B*	Frontotemporal lobar degeneration	CHMP2B	3	87,276,413	87,304,698	Less than 0
11	*DNAJB6*	Frontotemporal lobar degeneration	DNAJB6	7	157,129,692	157,210,133	Less than 0
12	*PRNPIP*	Other dementia	ERI3	1	44,686,742	44,821,315	Less than 0
13	*CSF1R*	Other dementia	CSF1R	5	149,432,854	149,492,935	Less than 0
14	*SNCA*	Parkinsonism	SNCA	4	90,645,250	90,759,447	Less than 0
15	*PARK2*	Parkinsonism	PARK2	6	161,768,590	163,148,834	1.4769
16	*UCHL1*	Parkinsonism	UCHL1	4	41,258,898	41,270,446	1.4769
17	*LRRK2*	Parkinsonism	LRRK2	12	40,618,813	40,763,087	Less than 0
18	*PINK1*	Parkinsonism	PINK1	1	20,959,948	20,978,004	Less than 0
19	*DJ1*	Parkinsonism	PARK7	1	8,021,714	8,045,342	Less than 0
20	*ATP13A2*	Parkinsonism	ATP13A2	1	17,312,453	17,338,467	Less than 0
21	*GIGYF2*	Parkinsonism	GIGYF2	2	233,562,015	233,725,287	Less than 0
22	*Omi/HTRA2*	Parkinsonism	HTRA2	2	74,756,532	74,760,683	Less than 0
23	*PLA2G6*	Parkinsonism	PLA2G6	22	38,507,502	38,577,857	Less than 0
24	*FBXO7*	Parkinsonism	FBXO7	22	32,870,707	32,894,818	Less than 0
25	*NUCKS1*	Parkinsonism	NUCKS1	1	205,681,947	205,719,372	Less than 0
26	*VPS35*	Parkinsonism	VPS35	16	46,693,589	46,723,144	Less than 0
27	*EIF4G*	Parkinsonism	EIF4G1	3	184,032,283	184,053,146	Less than 0
28	*DCTN1*	Parkinsonism	DCTN1	2	74,588,281	74,619,214	Less than 0
29	*POLG1*	Mitochondrial disease	POLG	15	89,859,536	89,878,026	Less than 0
30	*POLG2*	Mitochondrial disease	POLG2	17	62,473,902	62,493,184	Less than 0
31	*PEO1*	Mitochondrial disease	C10orf2	10	102,747,293	102,754,159	Less than 0
32	*ANT1*	Mitochondrial disease	SLC25A4	4	186,064,417	186,071,538	Less than 0
33	*STX6*	Progressive supranuclear palsy	STX6	1	180,942,164	180,992,074	Less than 0
34	*EIF2AK3*	Progressive supranuclear palsy	EIF2AK3	2	88,856,259	88,927,094	Less than 0
35	*MOBP*	Progressive supranuclear palsy	MOBP	3	39,509,064	39,570,988	Less than 0
36	*GBA*	Others	GBA	1	155,204,239	155,214,653	Less than 0
37	*NPC1*	Others	NPC1	18	21,086,148	21,166,581	Less than 0
38	*CYP27A1*	Others	CYP27A1	2	219,646,472	219,680,016	Less than 0
39	*TNPO1*	Others	TNPO1	5	72,112,418	72,210,215	Less than 0
40	*UBQLN1*	Others	UBQLN1	9	86,274,878	86,323,168	Less than 0
41	*UBQLN2*	Others	UBQLN2	X	56,590,025	56,593,443	Not analysed
42	*SQSTM1*	Others	SQSTM1	5	179,233,388	179,265,078	Less than 0
43	*ATXN1*	Others	ATXN1	6	16,299,343	16,761,721	Less than 0
44	*ATXN2*	Others	ATXN2	12	111,890,018	112,037,480	Less than 0
45	*ATXN3*	Others	ATXN3	14	92,524,896	92,572,965	1.4769
46	*ATN1*	Others	ATN1	12	7,033,626	7,051,484	Less than 0
47	*TBP*	Others	TBP	6	170,863,384	170,881,958	Less than 0
48	*GFAP*	Others	GFAP	17	42,982,994	42,992,920	Less than 0
49	*RGPD5*	Others	RGPD5	2	110,550,335	110,615,268	Less than 0
50	*RAN*	Others	RAN	12	131,356,617	131,362,220	Less than 0
51	*DNAJC6*	Others	DNAJC6	1	65,720,133	65,881,552	Less than 0
52	*NOP56*	Others	NOP56	20	2,633,178	2,639,039	Less than 0

*Chromosome numbers and positions are based on GRCh37.p13.

**The maximum LOD in the linkage analysis of this family was 1.4769.

Next, we selected 67 candidate genes for further analysis (see Supplemental Table S1). Among these genes, according to annotation analysis, we focused on the heterozygous missense mutation c.11596C>G, p.Pro3866Ala in *BSN* (Fig. 1B), which is expressed specifically in the central nervous system. This *BSN* mutation was detected only in the patients who presented with PSP-like syndrome. Co-segregation of this mutation with the symptoms was observed within the pedigree. This *BSN* mutation was not detected in blood samples from 100 healthy individuals. The frequency of the mutation was 0.00004170 in the Exome Aggregation Consortium (ExAC) database. In Case 2, immunohistochemical analysis of the BSN protein did not reveal loss of the protein in the hippocampus (CA1–4, subiculum), medial temporal lobe, or globus pallidus.

### Mutated rat BSN cDNA (c.11623C > G, p.Pro3875Ala) corresponding to the mutation (c.11596C > G, p.Pro3866Ala) found in the Japanese family with PSP-like syndrome may regulate tau

**Figure 3:**
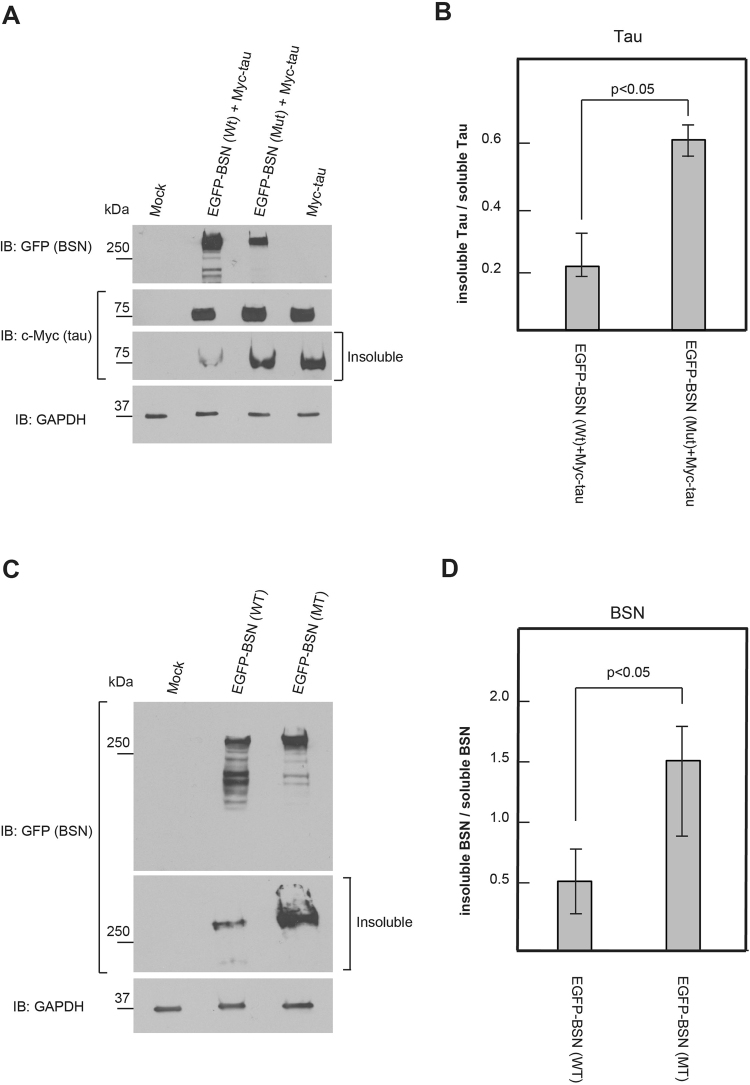
Western blot analysis of tau and wild-type BSN (BSN[Wt]) or mutated BSN (BSN[Mut]). (**A**) Protein assay of tau by overexpression of BSN(Wt) or BSN(Mut). HEK293T cells overexpressing cMyc-tagged tau, cMyc-tagged tau and EGFP-tagged BSN(Wt), and cMyc-tagged tau and EGFP-tagged BSN(Mut) were used. Western blot analysis of tau with cMyc-tagged tau and EGFP-tagged BSN(Wt), compared with HEK293T cells overexpressing cMyc-tagged tau, revealed the reduced accumulation of tau bands, especially in the insoluble fraction. However, in Western blot analysis of tau with cMyc-tagged tau and EGFP-tagged BSN(Mut), compared with HEK293T cells overexpressing cMyc-tagged tau, there was no reduction of tau bands in neither the insoluble nor soluble fraction. In this study, tau protein with 4 repeats was used. (**B**) Comparison of insoluble tau and soluble tau using ImageJ analysis showed that HEK293T cells overexpressing EGFP-tagged BSN(Mut) accumulated more insoluble tau than HEK293T cells overexpressing EGFP-tagged BSN(Wt). Data are means ± S.D. of values from three independent experiments. (**C**) HEK293T cells overexpressing EGFP-tagged BSN(Wt) and EGFP-tagged BSN(Mut) were used. Western blot analysis of BSN(Mut) compared with BSN(Wt) showed the accumulation of BSN in the insoluble fraction. GAPDH was used as an internal control. (**D**) Comparison of insoluble BSN and soluble BSN using ImageJ analysis showed the accumulation of BSN in the insoluble fraction and reduced degradation in HEK293T cells overexpressing EGFP-tagged BSN(Mut). Data are means ± S.D. of values from three independent experiments.

It is important to determine the pathogenic mechanism of the BSN mutation; therefore, we investigated the association between BSN and tau. Mutated rat BSN cDNA (c.11623C > G, p.Pro3875Ala), which corresponds to the mutation (c.11596C > G, p.Pro3866Ala) found in the Japanese family with PSP-like syndrome, was constructed. First, we transfected expression vectors encoding EGFP-tagged wild-type BSN (BSN[Wt]) or EGFP-tagged mutated BSN (BSN[Mut]) and cMyc-tagged tau into HEK293T cells and performed immunoblotting with anti-GFP and anti-c-Myc antibodies (Fig. 3A). BSN(Wt) and tau cDNA were used in a previous study^15,16^. Western blot analysis of tau with cMyc-tagged tau and EGFP-tagged BSN(Wt) revealed the reduced accumulation of tau bands, especially in the insoluble fraction, compared with HEK293T cells overexpressing cMyc-tagged tau. However, in Western blot analysis of tau with cMyc-tagged tau and EGFP-tagged BSN(Mut), compared with HEK293T cells overexpressing cMyc-tagged tau, there was no reduction of tau bands in either the insoluble or the soluble fraction (Fig. 3A). A comparison of insoluble tau and soluble tau using ImageJ analysis showed that HEK293T cells overexpressing EGFP-tagged BSN(Mut) accumulated much more insoluble tau than HEK293T cells overexpressing EGFP-tagged BSN(Wt)^17^ (Fig. 3B and Supplementary Fig. S5A and S5B).

Next, we examined the association between the BSN mutation and the insoluble and soluble fractions. We transfected expression vectors encoding EGFP-tagged BSN(Wt) or EGFP-tagged BSN(Mut) into HEK293T cells and performed immunoblotting with the anti-GFP antibody (Fig. 3C). Western blot analysis of BSN(Mut) compared with BSN(Wt) showed the accumulation of BSN in the insoluble fraction^17^ (Fig. 3D and Supplementary Fig. S5C and S5D).

### Absence of *BSN* mutations in the other pedigrees with PSP-like syndrome

In the four probands of four other pedigrees with PSP-like syndrome, no *BSN* mutations were detected. In one pedigree, a *TARDPB* mutation was identified^18^. In another pedigree, a *GRN* mutation was detected (c.352_354delAAC, p.N118del).

### Identification of novel *BSN* mutations in four cases among 41 patients with sporadic PSP-like syndrome

Next, *VCP*, *FUS*, *DNAJB6*, *CHMP2B*, *BSN*, *MAPT*, *C9ORF72*^19^, *GRN*, *DCTN1*, *TARDBP*, and *BSN* were analysed for mutations in 41 cases of sporadic PSP (sPSP)-like syndrome. As a result, six mutations in *DCTN1*, *GRN*, and *MAPT* were detected in five patients.

The *DCTN1* mutation c.3782 G > A (p.R1261Q) and the *GRN* mutation c.1498 G > A (p.V500I) were detected in both PSP-RS cases. The *GRN* mutation c.662 G > C (p.C221S) was detected in one PSP-PI case. The *DCTN1* mutation c.2686C > G (p.L896V) was detected in one PSP-FTD case. A double mutation (the *MAPT* mutation c.689 A > G (p.Q230R) and the *DCTN1* mutation c.2213 A > G (p.Q738R)) was detected in another PSP-FTD case. In the patients with these mutations, no *BSN* mutations were observed.

Moreover, in four cases among the other 36 patients, three novel missense *BSN* mutations were identified (Table 3). These missense mutations of the *BSN* gene consisted of a heterozygous base substitution in exon 3 (c.9436 C > T) in two patients, which resulted in a substitution of cysteine for arginine at codon 3146 (p.R3146C); a heterozygous base substitution in exon 3 (c.8564 C > T) in one patient, which resulted in a substitution of leucine for proline at codon 2855 (p.P2855L); and a heterozygous base substitution in exon 3 (c.10880 G > T) in one patient, which resulted in a substitution of valine for glycine at codon 3627 (p.G3627V). These *BSN* mutations are very rarely listed in databases of healthy subjects (1000 Genomes Project, Human Genetic Variation Database [HGVD], and ExAC). The application of SIFT, PolyPhen-2, or MutationTaster suggested that these mutations result in disease-causing changes in the protein (Table 3).

**Table 3:** Clinical features and *BSN* mutations of the patients with idiopathic PSP-like syndrome.

Case number	1	2	3	4
Disease type	PSP-PI	PSP-CBS	PSP-CBS	PSP-FTD
Mutation	c.9436 C > T (p.R3146C)	c.9436 C > T (p.R3146C)	c.8564 C > T (p.P2855L)	c.10880 G > T (p.G3627V)
Age at onset (years)	78	70	65	75
Age at examination (years)	83	76	72	76
Rigidity of the neck	−	+	−	+
Postural instability	++	++	−	+
Supranuclear palsy	±	+	±	±
Cognitive dysfunction	−	+	+	+
Atrophy of the temporal lobe	+	−	+	+
Atrophy of the brain stem	−	+	−	−
Hot cross bun sign	−	−	−	−
Other MRI findings	Abnormal pontine signal	None	None	Abnormal pontine signal
1000 Genomes	0.0002/1	0.0002/1	Unreported	T = 0.000
HGVD	0.003/1	0.003/1	Unreported	T = 0.002
ExAC	0.00003315	0.00003315	Unreported	0.0004181
SIFT	Damaging	Damaging	Damaging	Damaging
PolyPhen-2	Benign	Benign	Probably damaging	Probably damaging
MutationTaster	Disease causing	Disease causing	Disease causing	Disease causing

These variants were not detected among 200 chromosomes obtained from our 100 control subjects. Thus, the frequency of these alleles was significantly higher in PSP patients than in controls. Furthermore, these variants of the *BSN* gene were associated with a significantly increased risk of sPSP-like syndrome. Finally, *BSN* mutations were detected in ~10% of sPSP-like syndrome patients.

## Discussion

Here, we report the clinical and pathological features of a Japanese family with clinically identified PSP-like syndrome, in which we detected a putative disease-causing mutation in *BSN*. Moreover, we detected *BSN* mutations in four out of 41 patients with sPSP-like syndrome or CBS through gene analysis.

The three cases from the large pedigree exhibited cognitive decline and postural instability. Because postural instability and falls developed within one year after the onset of gait disturbance, we clinically diagnosed all of these cases as probable PSP according to the NINDS-PSP diagnostic criteria^11^. Moreover, all of the affected cases in this pedigree were classified as PSP-FTD^12^. However, these three familial cases did not exhibit typical PSP with respect to five points. First, forgetfulness was the initial symptom. Second, parkinsonism began 10 years after the onset of cognitive decline. Third, brain MRI showed the hot cross bun sign with brain stem atrophy. Fourth, medial temporal lobe atrophy was observed. Fifth, central apnoea and vocal cord paralysis were observed at an advanced stage. These findings indicated neurological and neuroradiological symptoms of both FTD and MSA. Therefore, these cases could be diagnosed as PSP-like syndrome. Furthermore, their pathological findings differed from those of previously reported PSP and PSP-like syndrome cases, including pallido-luysio-nigral atrophy.

PSP is associated with tau-positive cytoskeletal abnormalities in astrocytes and oligodendroglia as well as neurons. Tau-positive astrocytic structures (tuft-shaped astrocytes) have been studied to elucidate their significance in the neuropathological process of PSP^20^. In this case, tuft-shaped astrocytes were not detected. Furthermore, the observed tau accumulations contained both three-repeat and four-repeat tau; this pattern of tauopathy is more consistent with Alzheimer’s disease than with PSP^21^. Moreover, the loss of neurons in the hippocampus was severe. Beach *et al*. suggested the term ‘hippocampal sclerosis dementia with tauopathy’^22^. Additionally, Miki *et al*. reported a case of hippocampal sclerosis with round inclusions in the dentate gyrus that were positive for four-repeat tau but negative for three-repeat tau^23^. Our patients showed pathological features that had not previously been reported in those or any other studies. Thus, we considered genetic disorders that might present with a PSP-like phenotype and suspected an underlying genetic disorder on the basis of diagnostic algorithms^4^, and we identified a mutation in *BSN*.

Recently, ‘primary age-related tauopathy’ (PART) was described as a pathology commonly observed in the brains of aged individuals^24^. BSN protein levels are reportedly decreased significantly in the neuromuscular junctions of aged mice^25^. In the *BSN* mutation pedigree, the positive neurological findings for Case 3 (II-3) were so mild that PART may be a differential diagnosis. In the future, we should determine whether a subset of PART is caused by *BSN* mutations. Furthermore, in this novel family pedigree, the neurological symptoms of Case 3 (II-3) (i.e., the mother of Cases 1 and 2) were mild compared with those of her affected sons. Thus, genetic epistasis may contribute to the development of PSP and CBS^26^.

Although *BSN* wild-type and heterozygous mice survive normally, 50% of homozygous mutants die during the first six months after birth and display the typical posture of animals that have died from epileptic seizures^27^. BSN may be important for the normal function of the hippocampus, and further characterization of the identified *BSN* mutation may reveal a useful model for studying the effects of chronic changes in network activity^28^. The findings of severe hippocampal atrophy in the Japanese PSP-like family agree with those in the BSN mutant mice.

Furthermore, BSN is a presynaptic scaffolding protein that localizes specifically to CaV2.1 channels in active zones and is a major regulator of the molecular composition of presynaptic neurotransmitter release sites^29^. BSN and piccolo regulate ubiquitination and link presynaptic molecular dynamics to activity-regulated gene expression^30^. These previous studies could explain the pathogenic mechanism of *BSN* mutations and loss of function. A further study to determine whether these *BSN* mutations are associated with presynaptic molecular signal transduction should be considered.

The identification of homozygous and compound heterozygous mutations in a single gene in cases of familial neurodegenerative disease has been applied to identify genes associated with sporadic neurodegenerative diseases, such as MSA^31^. Single-nucleotide polymorphisms (SNPs) identified on the basis of SNP arrays from the international HapMap consortium and genetic variants detected in the 1000 Genomes Project serve as two references for genome-wide association studies^32,33^. The 1000 Genomes Project has elucidated the properties and distribution of common and rare variations, provided insights into the processes that shape genetic diversity, and advanced our understanding of disease biology^32^.

We performed a genome-wide study in 41 sPSP patients and 100 controls and identified *BSN* mutations. According to database analysis using the 1000 Genomes Project database^32^, HGVD, SIFT, PolyPhen-2, and MutationTaster, the four identified *BSN* mutations could generate disease-causing changes in the protein.

Furthermore, we detected an elevated frequency of *BSN* mutations in PSP-like syndrome patients. Although PSP must be diagnosed on the basis of neurological and pathological findings, most individuals with PSP-like syndrome do not undergo a pathological examination. Thus, the presence of a *BSN* mutation could be used to establish one clinical disease category.

Our results showed that the neurodegenerative disorder in the proband’s pedigree is a novel tauopathy, differing from known dementia and parkinsonism syndromes, including PSP. As the *BSN* mutation c.11596C > G, p.Pro3866Ala was detected only in affected cases in the pedigree and the *BSN* mutations observed in familial and sporadic cases are very rare in databases of healthy controls, these mutations may be considered to contribute to this PSP-like syndrome.

BSN is a large protein that is over 400 kDa, and it is technically very difficult to mutate *BSN* cDNA, which is why we compared the brain of the patient harbouring a *BSN* mutation with a normal brain to examine the pathogenic role of this mutation. Unexpectedly, the accumulation of tau was observed very clearly in the patient’s brain compared with the control brain (see Supplementary Fig. S4). This result made us certain that the case was very important and demonstrated a new tauopathy. This finding led us to attempt to identify the molecular mechanism of the tauopathy caused by the *BSN* mutation. Interestingly, the *BSN* mutation in the Japanese family with PSP-like syndrome may regulate the accumulation of tau (Fig. 3A and B); moreover, it may regulate the degradation and expression pattern of BSN protein (Fig. 3C and D). Although causative gene mutations for some neurodegenerative disorders have been reported without describing the underlying molecular mechanisms^34^, the present study indicated that the identified *BSN* mutation had a possible role in the pathogenesis of PSP-like syndrome through tau accumulation. BSN protein reportedly regulates the degradation of multiple presynaptic proteins through ubiquitination and autophagy^35,36^. Regarding tauopathy, some genetic mutations involved in tauopathy are not directly associated with the tau protein. For example, neurofibrillary tangles composed of aggregates of the highly soluble protein tau are present in Niemann-Pick type C cases^37^. Additionally, several genetic mutations outside the *MAPT* gene are reportedly associated with tauopathy^38^.

Regarding *BSN* gene mutation, another cohort study of patients with sporadic PSP syndrome and further functional investigations are needed to understand how mutant BSN might participate in the pathophysiology of PSP-like syndrome.

In conclusion, the present study demonstrated the clinical and pathological features of a PSP-like syndrome due to *BSN* mutations. Additional patients with clinical PSP-like phenotypes, CBS, MSA, and hippocampal sclerosis dementia should be identified to determine whether *BSN* mutations are present.

## Methods

### Patients and families

This study was approved by the Medical Ethics Committee of the Hokkaido University Graduate School of Medicine (13-012). All methods were in accordance with relevant guidelines and regulations. Written informed consent was obtained from all participants. The PSP phenotype was clinically diagnosed using the NINDS international diagnostic criteria for PSP: (1) age at onset >40 years and (2) the presence of a gradually progressive disorder, combined with (3) slow vertical saccades or supranuclear gaze palsy and (4) early postural instability and falls during the first year of the disease^1,11^. Clinical data and biological samples were collected from all patients. The genealogies of the families were reconstructed, and clinical data and biological samples from relatives were collected whenever possible. We defined phenotypes that fulfilled the above PSP diagnostic criteria but were not diagnosed pathologically as ‘PSP-like syndrome’. We assessed patients from a pedigree with dominant PSP-like syndrome (Fig. 1A) that included three affected individuals (II-3, III-1, and III-2) and four unaffected individuals (II-5, II-6, III-3, and III-4); four probands from four pedigrees with PSP-like syndrome in whom a family history of PSP-like syndrome was identified only through medical interviews; and 41 cases of sPSP-like syndrome, which were classified as 18 cases of PSP Richardson’s syndrome (PSP-RS), 12 cases of PSP-CBS, nine PSP-FTD, and two cases of PSP-postural instability (PSP-PI^12^).

### Neuropathological examinations

The brain of Case 2 (III-1) was fixed with 10% buffered formalin and embedded in paraffin. For routine histological examinations, 4-µm-thick sections from multiple cortical and subcortical regions were stained with haematoxylin and eosin or via the Klüver-Barrera and Gallyas-Braak methods. We also examined paraffin-embedded sections immunohistochemically using the primary antibodies listed in Supplemental Table S2. Antigen retrieval was performed with EnVision FLEX TRS high pH for phosphorylated α-synuclein and BSN; with citrate buffer (pH 6.0) for phosphorylated tau, three-repeat tau, four-repeat tau, β-amyloid, and phosphorylated TDP-43; and with formic acid for polyglutamine.

Paraffin sections from the hippocampus of Case 2 (III-1) were processed for double-labelling immunofluorescence. Deparaffinized sections were incubated overnight at 4 °C with a mixture of anti-three-repeat tau (RD3; Millipore; 1:50) and anti-four-repeat tau antibodies (Cosmo Bio Co., LTD.; 1:300). The sections were then rinsed and incubated with anti-rabbit IgG tagged with Alexa Fluor 488 (Invitrogen, Carlsbad, CA, USA; 1:1,000) or anti-mouse IgG tagged with Alexa Fluor 594 (Invitrogen; 1:1,000) for 2 h at 4 °C. The sections were subsequently mounted with Vectashield (Vector Laboratories, Inc., Burlingame, CA, USA) and examined using a confocal microscope (EZ-Ci; Nikon, Tokyo, Japan).

### Genetic methods

#### Mapping and SNV/indel calling

Adapter sequences were removed from reads with cutadapt (v1.2.1). After quality control, the reads were mapped to the reference human genome (hg19) using BWA (ver. 0.6.2). The mapping result was corrected using Picard (ver. 1.73) for the removal of duplicates and with GATK (ver. 1.6-13) for local alignment and quality score recalibration. Single-nucleotide variant (SNV) and insertion/deletion (indel) calling were performed via multisample calling using GATK and were filtered to coordinates based on passage through the VQSR filter and a variant call quality score ≥ 30. The annotation of SNVs and indels was based on dbSNP138, CCDS (NCBI, Aug 2012), RefSeq (UCSC Genome Browser, Jul 2013), Gencode (UCSC Genome Browser, ver. 17), 1000 Genomes (Oct 2012), and HGVD (ver. 1.41). Variants were further filtered according to the following criteria: predicted frameshift, nonsense, read-through, missense, deletion, insertion, or indel functions.

**Figure 4:**
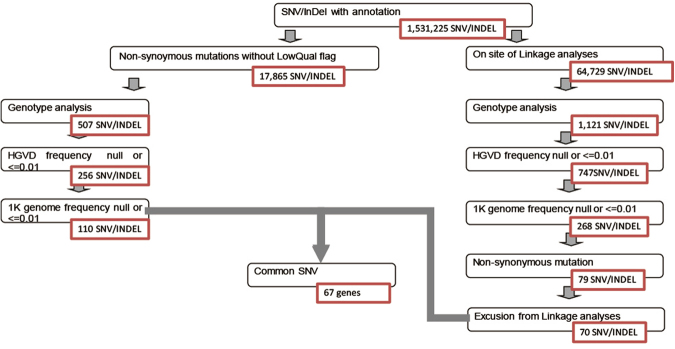
Strategies for the identification of single nucleotide variants as final candidates.

The annotation of SNVs and indels was based on dbSNP138, CCDS (NCBI, Release 12), RefSeq (UCSC Genome Browser, Jul 2013), Gencode (UCSC Genome Browser, ver. 17), 1000 Genomes, HGVD, and ExAC. Variants were filtered according to the following criteria, as shown in Fig. 4:

Non-synonymous mutations: variants with predicted frameshift, nonsense, read-through, missense, deletion, insertion, or indel functions were selected as candidates. Variants marked with a LowQual flag by GATK were removed for quality filtering.

On-site linkage analyses: mutations with LOD scores > 0 were chosen as candidates.

Genotype analysis: SNVs observed in affected family members but not in unaffected members were chosen from the candidates.

Indels with HGVD frequency null or ≤0.01 and 1000 Genomes frequency null or ≤0.01, as well as SNVs with an allelic frequency <0.01 or null in HGVD and the 1000 Genomes Project, were selected.

Exclusion from linkage analyses: Mendelian inheritance types for which the LOD score did not show a clear pattern were excluded from the results.

Finally, SNVs found in all samples were identified as candidates.

### Genotyping and linkage analysis in the family and four additional probands

SNP genotyping was conducted using a HumanOmniExpressExome-8 BeadChip (Illumina, CA, USA) according to the manufacturer’s protocol (Riken Genesis Co., Ltd. (Tokyo, Japan)). The SNP genotype data were used for parametric linkage analysis to define a disease-related locus. The quality of the SNP genotype data was evaluated using PLINK software^39^. An average call rate of >99% and an estimated IBD value of <0.8 were required for linkage analysis. All samples satisfied these criteria and were used for this analysis. We excluded 215 SNPs owing to Mendelian inconsistencies. In the family with PSP-like syndrome, genotyping and linkage analysis of three affected individuals (II-3, III-1, and III-2) and four unaffected individuals (II-5, II-6, III-3, and III-4) were performed.

In the family with PSP-like syndrome, the SNPs that were utilized for linkage analysis were selected on the basis of the following criteria: (i) select one tag SNP from every region with strong linkage disequilibrium (r^2^ ≥ 0.8), and (ii) select one tag SNP per 0.25 cM (250,000 bp)^40^. Finally, 1000 SNPs were selected from each chromosome. Linkage analysis was executed on the basis of three types of Mendelian inheritance. The penetrance of each pattern was as follows (‘a’ is the risk allele):

$$\begin{array}{c}\begin{array}{l}{\rm{Autosomal}}\,\mathrm{dominant}:{\rm{a}}{\rm{/}}{\rm{a}}=0.99,\,{\rm{a}}{\rm{/}}{\rm{A}}=0.99,\,{\rm{A}}{\rm{/}}{\rm{A}}=0.01\\ {\rm{Autosomal}}\,{\rm{additive}}:{\rm{a}}{\rm{/}}{\rm{a}}=0.99,\,{\rm{a}}{\rm{/}}{\rm{A}}=0.5,\,{\rm{A}}{\rm{/}}{\rm{A}}=0.01\end{array}\\ {\rm{Autosomal}}\,{\rm{recessive}}:{\rm{a}}{\rm{/}}{\rm{a}}=0.99,\,{\rm{a}}{\rm{/}}{\rm{A}}=0.01,\,{\rm{A}}{\rm{/}}{\rm{A}}=0.01\end{array}$$

The minor allele frequency for a disease-related locus was set to 0.0005. Linkage analysis was performed using MERLIN software^41^, and LOD scores were calculated.

### Target selection and sequencing

Genomic DNA was extracted from the white blood cells of individuals with PSP-like syndrome via standard protocols associated with the Viogene Blood & Tissue Genomic DNA Extraction Miniprep System (Gentaur, Kampenhout, Belgium) and was then submitted to Riken Genesis Co., Ltd. (Tokyo, Japan) for exome sequencing and bioinformatics analysis. Genomic DNA was sheared into approximately 300 bp fragments and used to generate a library for multiplexed paired-end sequencing (Illumina). The constructed library was hybridized to biotinylated cRNA oligonucleotide baits from the SureSelect Human All Exon V5 Kit (Agilent Technologies, Santa Clara, CA, USA) for exome capture. Targeted sequences were purified using magnetic beads, amplified, and sequenced on the Illumina HiSeq. 2000 platform in the paired-end 100 bp configuration. In addition, genetic analyses of the expansion of *NOP56*^42^, *C9ORF72*^19^, the causative genes of various spinocerebellar ataxias^43^, and the whole mitochondrial genome^44^ were conducted.

### CNV analysis

CNV analysis was conducted for seven samples in the family pedigree (Fig. 1A). The Illumina cnvPartition v3.2.0 CNV Analysis Plug-in for GenomeStudio Software was used to calculate CNV and confidence values for all chromosomes. The default value was adopted for each parameter of the plug-in. The CNV regions were listed using the Illumina CNV Region Report Plug-in v2.1.1. The distributions of the CNV value, log R ratio, and B allele frequency were visualized using the Illumina Genome Viewer embedded in GenomeStudio Software to confirm whether the detected CNVs in the genes with high LOD scores were false positives or true negatives.

### Genotyping in 41 sporadic cases

An amplicon sequencing panel targeting all exons of 10 genes (*VCP*, *FUS*, *DNAJB6*, *CHMP2B*, *BSN*, *MAPT*, *C9ORF72*, *GRN*, *DCTN1*, and *TARDBP*) was designed using a GeneRead Custom Panel (CNGHS-00689 × −494; QIAGEN, Tokyo, Japan). Genomic DNA was extracted from peripheral blood mononuclear cells derived from patients using a QIAamp DNA Mini Kit (QIAGEN). The quality of the genomic DNA was assessed using a Qubit dsDNA BR Assay Kit (Invitrogen), a Qubit Fluorometer 2.0 (Invitrogen), and a GeneRead DNA QuantiMIZE Assay Kit (QIAGEN). A GeneRead DNAseq Targeted Panel V2 (QIAGEN) was used for library preparation with 30–100 ng of genomic DNA, following the manufacturer’s instructions. The quality of the libraries was assessed using an Agilent 2100 Bioanalyzer (Agilent), an Agilent DNA 1000 Kit (Agilent), and a GeneRead Library Quant Kit (QIAGEN). The libraries were sequenced using the Illumina MiSeq platform to produce 150 bp paired-end reads. The raw read data obtained from amplicon sequencing were processed using online analytical resources from the GeneRead DNAseq Variant Calling Service (http://ngsdataanalysis.sabiosciences.com/NGS2/) for the analysis of mutations and CNVs. The annotation of SNVs was based on 1000 Genomes, HGVD, and ExAC.

#### Sanger sequencing

Sanger sequencing was performed to confirm that the mutations identified via exome sequencing segregated with the disease. For the *BSN* mutations, PCR was performed using GoTaq Green Master Mix (Promega, Tokyo, Japan) and NucleoSpin Gel and PCR Clean-up (Takara Bio, Kusatsu, Japan), with the primer sequences and PCR conditions shown in Supplemental Tables S3 and S4. Each *BSN* mutation was analysed in 100 healthy Japanese subjects.

#### Cell culture and immunoblot analysis

HEK293T cells were maintained in Dulbecco’s modified Eagle’s medium (Sigma-Aldrich) supplemented with penicillin (100 units/mL), streptomycin (100 μg/mL) and 10% heat-inactivated foetal calf serum (Invitrogen, Paisley, UK) at 37 °C in a humidified incubator with a 5% CO_2_ atmosphere. HEK293T cells were transfected with FuGENE HD (Promega) and then cultured for 48 h. Immunoblot analysis was performed as reported previously^45^. Mutated rat BSN cDNA (c.11623C > G, p.Pro3875Ala) (BSN[Mut]), which corresponds to the mutation (c.11596C > G, p.Pro3866Ala) found in the Japanese family with PSP-like syndrome, was constructed with a QuikChange mutagenesis kit using KOD-plus-neo (KOD-401; TOYOBO) (see Supplementary Table S5). BSN(Wt) and tau cDNA were used in previous studies^15,16^. Student’s *t*-test was used to determine the statistical significance of experimental data.

## Electronic supplementary material

Supplementary data
