# Supplementary material for: Mutations in bassoon in individuals with familial and sporadic progressive supranuclear palsy-like syndrome
Source: Sci Rep. 2018 Jan 16;8:819. doi: 10.1038/s41598-018-19198-0 (PMC5770378; doi:10.1038/s41598-018-19198-0)
Supplement: Supplementary file 1 — Supplementary data [file 41598_2018_19198_MOESM1_ESM.pdf]

Supplementary Information

Mutations in bassoon in individuals with familial and sporadic progressive supranuclear palsy-like syndrome

Ichiro Yabe[1*], Hiroaki Yaguchi[1,4], Yasutaka Kato[2,11], Yasuo Miki[3], Hidehisa Takahashi[4], Satoshi Tanikawa[2], Shinichi Shirai[1], Ikuko Takahashi[1], Mari Kimura[1], Yuka Hama[1], Masaaki Matsushima[1], Shinsuke Fujioka[5], Takahiro Kano[1], Masashi Watanabe[4], Shin Nakagawa[6], Yasuyuki Kunieda[7], Yoshio Ikeda[8], Masato Hasegawa[9], Hiroshi Nishihara[2,12], Toshihisa Ohtsuka[10], Shinya Tanaka[2,13], Yoshio Tsuboi[5], Shigetsugu Hatakeyama[4], Koichi Wakabayashi[3], Hidenao Sasaki[1]

1. Department of Neurology, Faculty of Medicine and Graduate School of Medicine, Hokkaido University, Sapporo, Japan
2. Department of Cancer Pathology, Faculty of Medicine and Graduate School of Medicine, Hokkaido University, Sapporo, Japan
3. Department of Neuropathology, Hirosaki University Graduate School of Medicine, Hirosaki, Japan
4. Department of Biochemistry, Faculty of Medicine and Graduate School of Medicine, Hokkaido University, Sapporo, Japan
5. Department of Neurology, Fukuoka University School of Medicine, Fukuoka, Japan
6. Department of Psychiatry, Faculty of Medicine and Graduate School of Medicine, Hokkaido University, Sapporo, Japan
7. Wakkanai City Hospital, Wakkanai, Japan
8. Department of Neurology, Gunma University Graduate School of Medicine, Maebashi,

Japan

9. Department of Dementia and Higher Brain Function, Tokyo Metropolitan Institute of Medical Science, Tokyo, Japan
10. Department of Biochemistry, Faculty of Medicine / Graduate School of Medicine, University of Yamanashi, Chuo, Japan
11. Laboratory of Oncology, Hokuto Hospital, Obihiro, Japan
12. Division of Clinical Cancer Genomics, Cancer Center, Keio University School of Medicine, Tokyo, Japan
13. Global Station for Soft Matter, Global Institution for Collaborative Research and Education, Hokkaido University, Sapporo, Japan

*Correspondence to yabe@med.hokudai.ac.jp

Table of contents

Supplemental Fig. S1. Co-localization of three-repeat tau and four-repeat tau in neurofibrillary tangles in the dentate gyrus

Supplemental Fig. S2. Western blot analysis of tau in a freshly frozen sample of the frontal cortex

Supplemental Fig. S3. Western blot analysis of BSN in a freshly frozen sample of the frontal cortex

Supplemental Fig. S4. Western blot tau analysis of a fresh frozen sample of frontal cortex from Case2 compared with a normal control brain

Supplemental Fig. S5. Western blot analysis of tau and wild-type BSN (BSN[Wt]) or mutated BSN (BSN[Mut])

Supplemental Fig. S6. Raw blot images of figures

Supplemental Table S1. Sixty-seven candidate genes

Supplemental Table S2. Primary antibodies used in this study

Supplemental Table S3. Primers used in this study

Supplemental Table S4. PCR method used in this study

Supplemental Table S5. PCR primers for mutated rat bassoon cDNA

Supplemental Fig. S1.

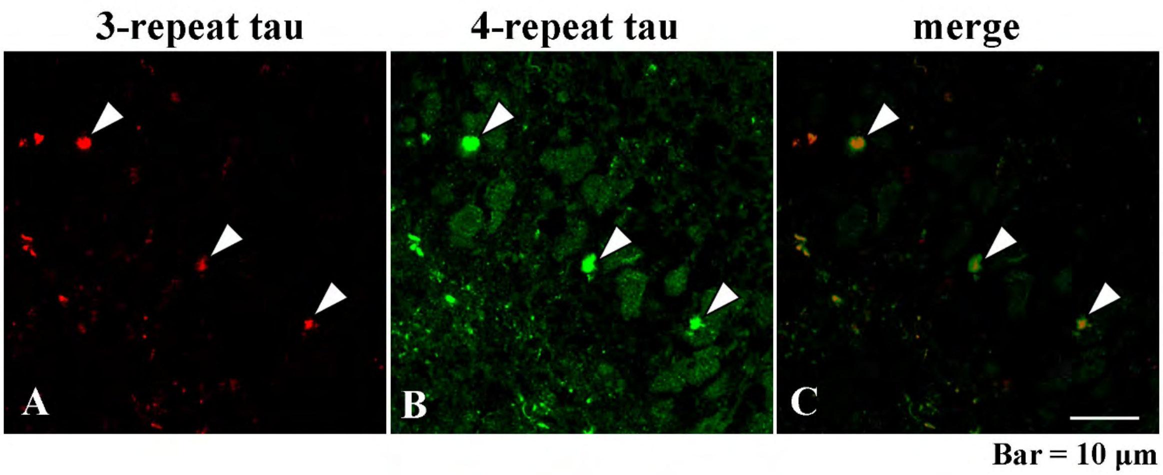

Supplemental Fig. S1. Co-localization of three-repeat tau and four-repeat tau in neurofibrillary tangles in the dentate gyrus

Double-labeling immunofluorescence demonstrating the co-localization of three-repeat tau and four-repeat tau in neurofibrillary tangles in the dentate gyrus (arrowheads) (A-C). Three-repeat tau appears *red*, and four-repeat-tau appears *green*. Bar = 10 µm.

Supplemental Fig. S2

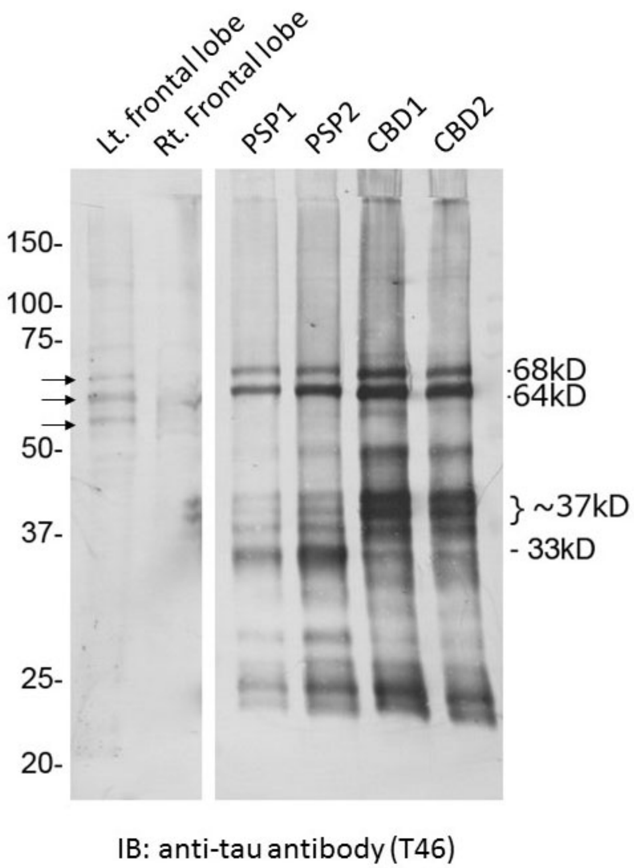

Supplemental Fig. S2. Western blot analysis of tau in a freshly frozen sample of the frontal cortex.

Western blot analysis of tau, performed according to a previously reported method[1], revealed phosphorylated triplet tau bands (60, 64, and 68 kDa) (arrow) that were similar to those observed in Alzheimer's disease. From left to right: left frontal lobe and right frontal lobe of case 2 and 4 disease controls (2 PSP and 2 CBD cases).

Supplemental Fig. S3

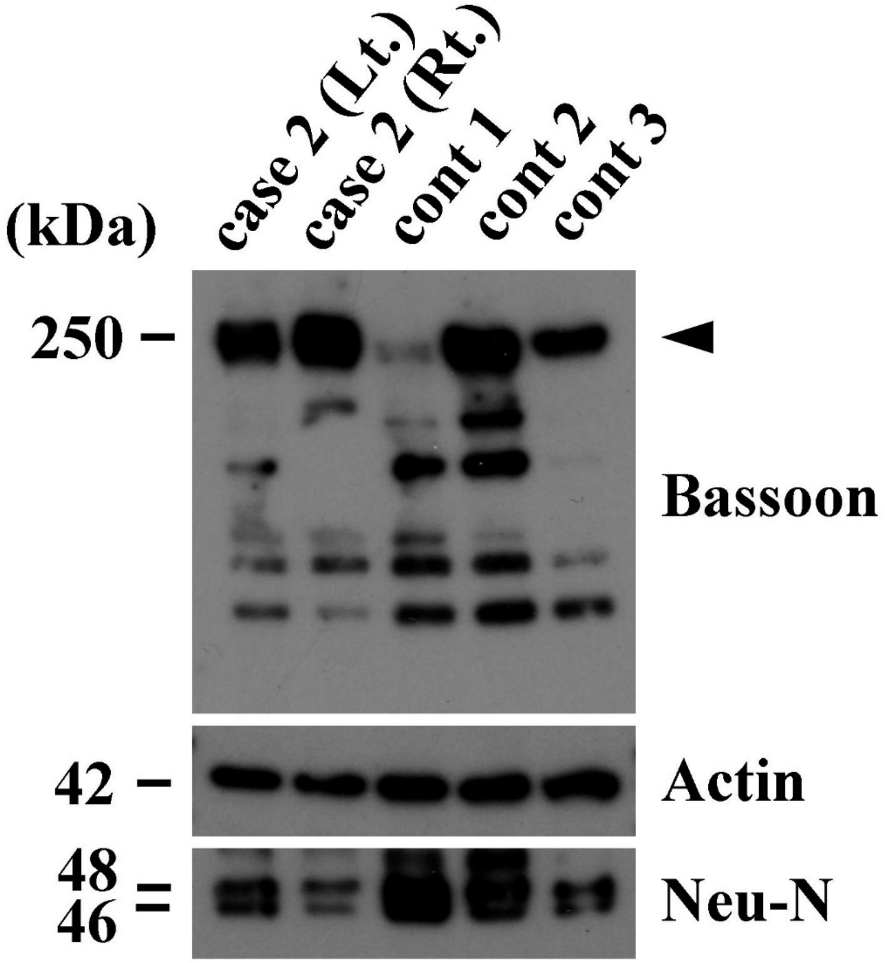

Supplemental Fig. S3. Western blot analysis of BSN in a freshly frozen sample of the frontal cortex.

Western blot analysis of the BSN protein using a BSN antibody (SAP7F407; Abcam, Cambridge, UK; 1:150) did not reveal a decrease in this patient. From left to right: left (Lt) frontal lobe and right (Rt) frontal lobe of case 2 and 3 disease controls (cont 1-3). Western blot analysis was performed according to a previously reported method [2].

Supplemental Fig. S4

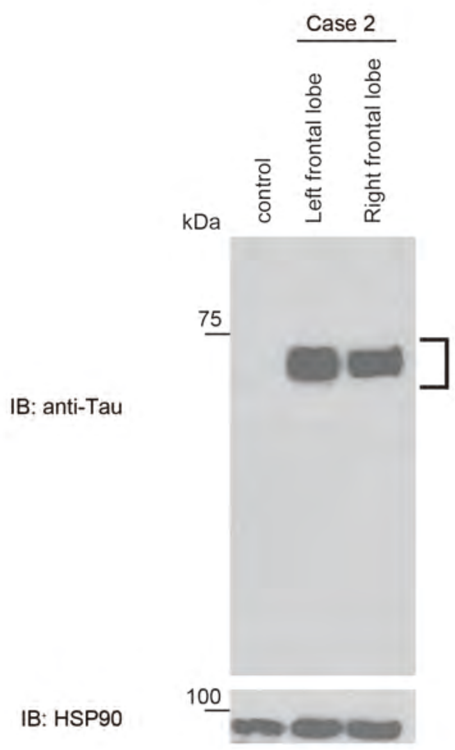

Supplemental Fig. S4. Western blot tau analysis of a fresh frozen sample of frontal cortex from Case2 compared with a normal control brain.

Western blot analysis of tau, compared with a normal control brain, revealed an accumulation of tau bands in the brain of case 2. HSP90 was used as an internal control.

Supplemental Fig. S5

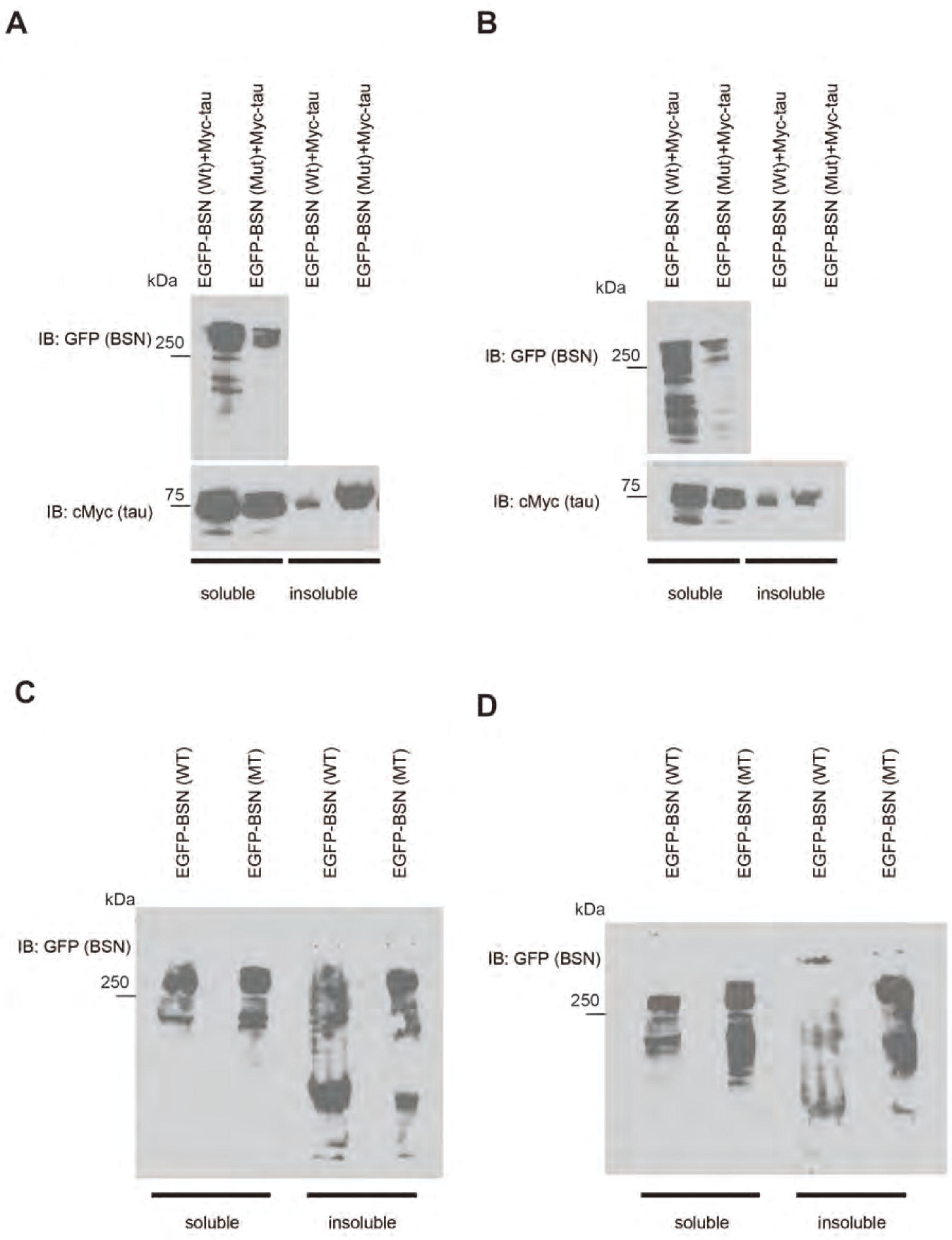

Supplemental Fig. S5. Western blot analysis of tau and wild-type BSN (BSN[Wt]) or mutated BSN (BSN[Mut]).

(A and B) Protein assay of tau following overexpression of BSN(Wt) or BSN(Mut). HEK293T cells overexpressing cMyc-tagged tau and EGFP-tagged BSN(Wt), and cMyc-tagged tau and EGFP-tagged BSN(Mut) were used. Western blot analysis of tau with cMyc-tagged tau and EGFP-tagged BSN(Mt), compared with HEK293T cells overexpressing cMyc-tagged tau and EGFP-tagged BSN(Wt), revealed the reduced accumulation of tau bands in the insoluble fraction. In this study, tau protein with 4 repeats was used.

(C and D) HEK293T cells overexpressing EGFP-tagged BSN(Wt) and EGFP-tagged BSN(Mut) were used. Western blot analysis of BSN(Mut) compared with BSN(Wt) showed the accumulation of BSN in the insoluble fraction.

Supplemental Fig. S6

A)

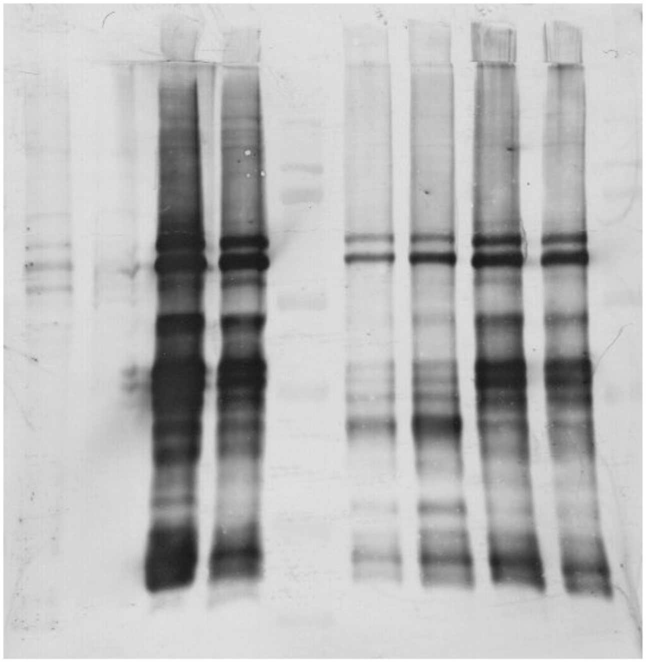

B)

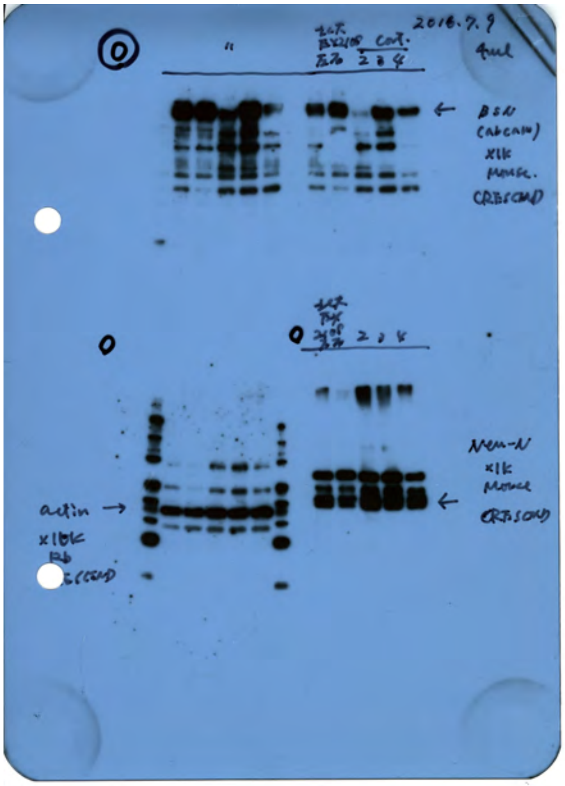

2018.7.9
4ul
BSN
(abcam)
x1k
Mouse.
CRBSOMD
Neu-N
x1k
Mouse
CRBSOMD
actin
x10k

C)

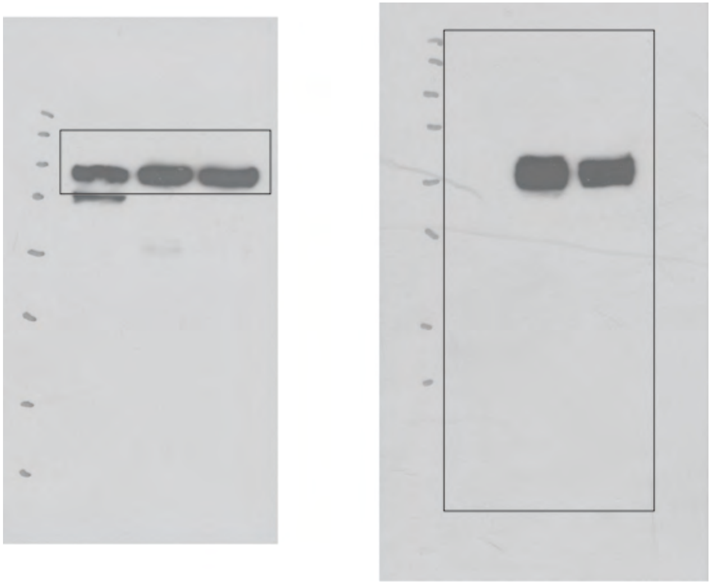

D)

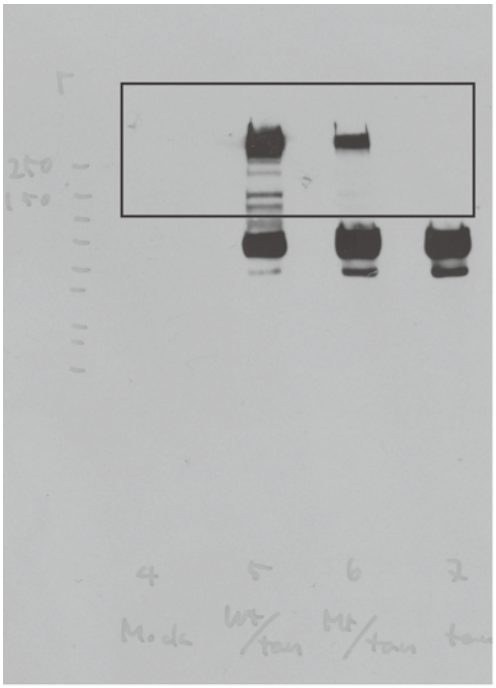
250
150
4 5 6 7
Mock Wt/tau Mt/tau tau

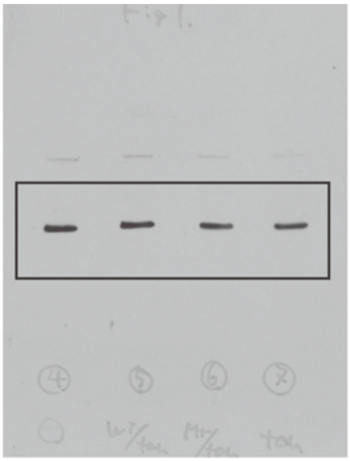
4 5 6 7
Wt/tau Mt/tau tau

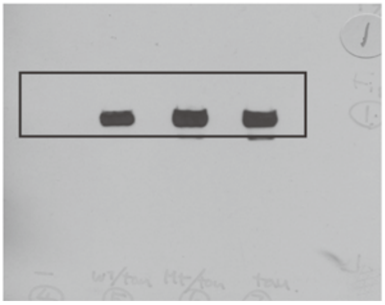
Wt/tau Mt/tau tau

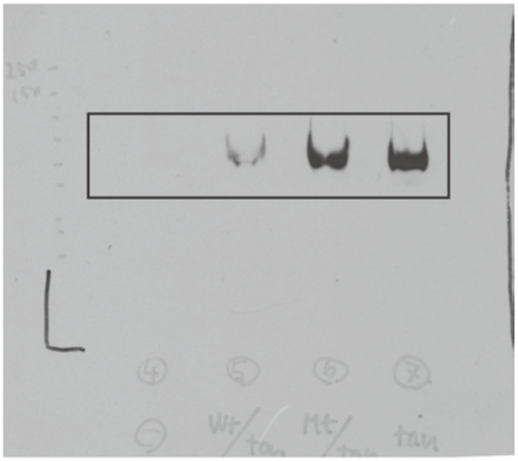
4 5 6 7
Wt/tau Mt/tau tau

E)

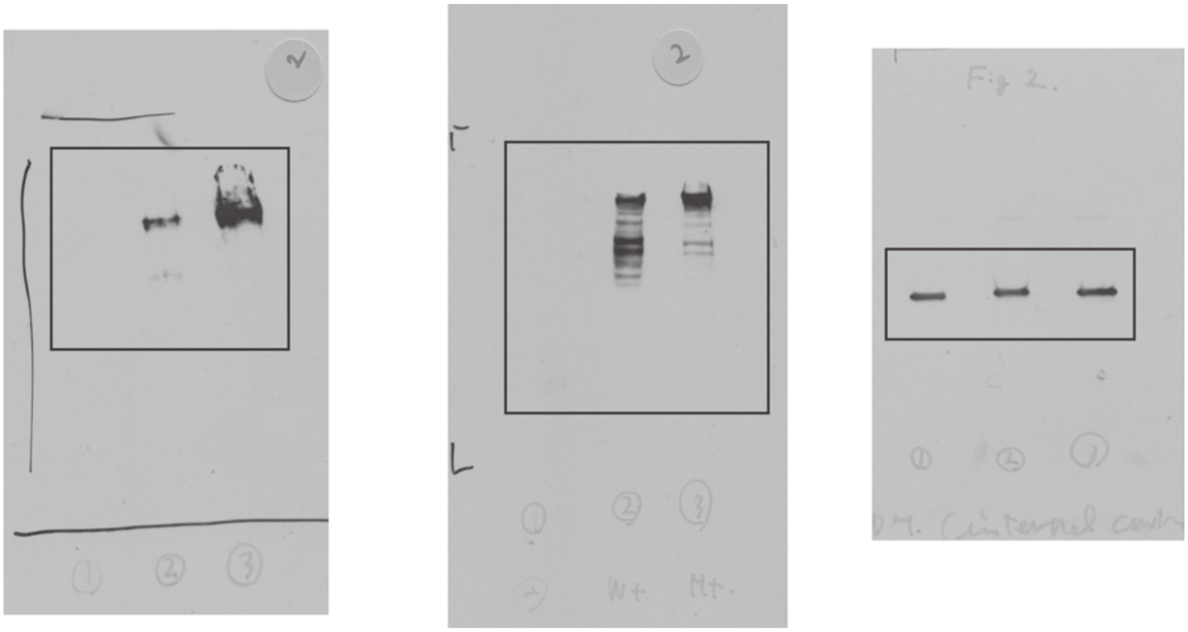

F)

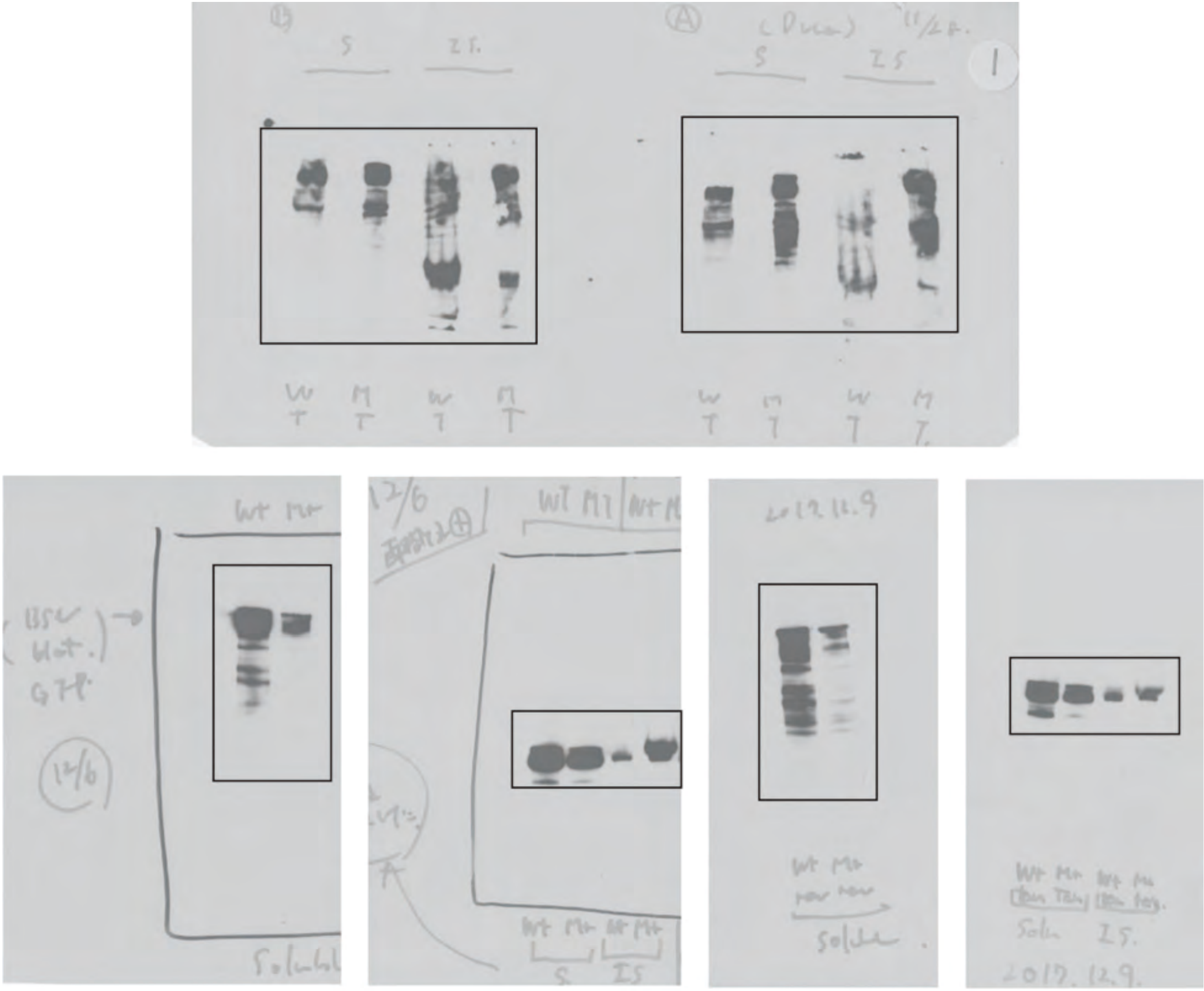

Supplemental Fig. S6. Raw blot images of figures.

A) Raw blot image of supplemental Fig. S2

The grouping of blots cropped from different parts of the same blots and same exposure.

B) Raw blot image of supplemental Fig. S3

The grouping of blots cropped from different parts of the same blots and same exposure.

C) Raw blot images of supplemental Fig. S4

The grouping of blots cropped from different parts of the different gel and different exposure.

D) Raw blot images of Fig. 3A and B

The grouping of blots cropped from different parts of the different gel and different exposure.

E) Raw blot images of Fig. 3C and D

The grouping of blots cropped from different parts of the different gel and different exposure.

F) Raw blot images of Fig. 3C and supplemental Fig. S5

The grouping of blots cropped from different parts of different gels and different exposures.

Supplemental Table S1. Sixty-seven candidate genes.

No	Gene
1	*SLC35E2,RP1-283E3.8*
2	*AGO3,RP4-665N4.8*
3	*ADAR*
4	*DYSF*
5	*ANKRD36*
6	*SEPT10,SOWAHC*
7	*ORC4*
8	*CSRNP3*
9	*STT3B*
10	*TMEM158*
11	*MAP4*
12	*COL7A1*
13	*NDUFAF3,DALRD3,MIR191*
14	*BSN*
15	*ETV5,ETV5-AS1*
16	*THAP9,LIN54*
17	*NKX6-1*
18	*DSPP,RP11-742B18.1*
19	*LARS*
20	*CTB-78H18.1*
21	*HLA-B*
22	*DOM3Z,STK19*
23	*FGD2*
24	*SOGA3*
25	*HGC6.3,RP3-470B24.5*
26	*FAM120B*
27	*NACAD*
28	*MUC12*
29	*KRBA1*
30	*FAM21A,FAM21B*
31	*ADM,CAND1.11*
32	*CAPN5*
33	*GAB2*
34	*CEP164*
35	*WNK1*
36	*PRMT8*
37	*KRT6B*
38	*OR6C65*

39	*ORC76*
40	*MYRFL*
41	*ZFC3H1*
42	*LRRIQ1*
43	*DEPDC4,SCYL2*
44	*WDFY2*
45	*PCCA*
46	*TMCO3*
47	*HOMEZ,RP11-124D2.6*
48	*PLEKHH1*
49	*BTBD7*
50	*CCNK*
51	*AHNAK2*
52	*FMN1*
53	*GOLGA8B,GOLGA8A*
54	*TFAP4*
55	*CBFA2T3,RP11-830F9.6*
56	*SEZ6,PIPOX*
57	*CCL4L1,CCL4L2*
58	*RAB40B*
59	*POTEC*
60	*SETBP1*
61	*ZBTB7C*
62	*FUT3*
63	*NDUFA11,FUT5,AC024592.12*
64	*DMKN*
65	*DIDO1*
66	*LSS,AP001468.1*
67	*TPST2*

Supplemental Table S2. Primary antibodies.

No	Antibody
1	phosphorylated tau (AT8; Thermo Scientific, Waltham, MA, USA; 1:200)
2	phosphorylated α-synuclein (pSyn#64; Wako, Tokyo, Japan; 1:1,000)
3	bassoon (SAP7F407; Abcam, Cambridge, UK; 1:150)
4	polyglutamine (5TF1-1C2; Merck Millipore, Darmstadt, Germany; 1:10,000)
5	rabbit polyclonal anti-β-amyloid (4G8; BioLegend, San Diego, CA, USA; 1:500)
6	phosphorylated TDP-43 (pS409/410-2; Cosmo Bio, Tokyo, Japan; 1:5,000)
7	FUS (SIGMA, St. Louis, MO, USA; 1:2,000)
8	three-repeat tau (RD3; Millipore, Billerica, USA, monoclonal; 1:500)
9	four-repeat tau (RD4; Millipore; 1:100)
10	tau monoclonal antibody (T46) (1:1000)
11	HSP90 (BD, 610418; 1:2000)
12	GAPDH (Ambion; 1:2000)
13	c-Myc (Wako; 1:1000)
14	GFP (Wako; 1:1000)

Supplemental Table S3. Primers used in this study.

Primer #	Chrom	Pos GRCh37			Mutation	dbSNP ID	Forward			Reverse			Product size (bp)	Position
			-200 bp	+200 bp			Sequence (5′ → 3′)	LEN	Tm	Sequence (5′ → 3′)	LEN	Tm		
1	3	49698714	49698514	49698914	c.9436 C>T, p.R3146C	rs201112949	AGGCCACTATGCAGGCCAAA	20	62.2	GGACCTTGCCCTGCTCATAG	20	60.2	239	chr3:49698623+49698861
2	3	49695553	49695353	49695753	c.8564 C>T, p.P2855L		GCTGAACAAAGCTCACGTGAG	21	60.1	CCTGTTCCCATACCTGGCTAC	21	59.9	206	chr3:49695437+49695642
3	3	49700471	49700271	49700671	c.10880 G>T, p.G3627V	rs200611323	ACGGACTGGTTTGATAAGCCC	21	60.3	CATAGCTGGAGCAGAGCTGG	20	60.2	333	chr3:49700332+49700664
4	3	49701307	49701107	49701507	c.11596C>G, p.P3866A		GTTCTGTGTTGCAGCCACGG	20	62.4	TTAGTGAGGGCATGCAGTGTG	21	60.6	220	chr3:49701206+49701425

Abbreviations: Chrom, chromosome; LEN, length; Tm, melting temperature; bp, base pairs; Pos, position

Supplemental Table S4. PCR method used in this study.

gDNA (25 ng/μL)	1 μL
GoTaq Green Master Mix	12.5 μL
Primer Mix (10 μM)	2.5 μL
DDW	9 μL
Total	25 μL

GoTaq Green Master Mix (Promega #M7122)
Primer Mix ＝ Forward+Reverse (each 5 μM)

95°C		2 min	
95°C	30 s		×32
60°C	30 s		
72°C	30 s		
72°C		5 min	
4°C		∞	

Supplemental Table S5. PCR primers for mutated rat bassoon cDNA

Forward	5′-CAGTCAGCTCCAGGAGCTGCAGGGGCGAAGACT-3′
Reverse	5′-AGTCTTCGCCCCTGCAGCTCCTGGAGCTGACTG-3′

References

1. Taniguchi-Watanabe, S. *et al.* Biochemical classification of tauopathies by immunoblot, protein sequence and mass spectrometric analyses of sarkosyl-insoluble and trypsin-resistant tau. *Acta Neuropathol.* **131**, 267-280 (2016).
2. Zhang, H. X., Tanji, K., Mori, F. & Wakabayashi, K. Epitope mapping of 2E2-D3, a monoclonal antibody directed against human TDP-43. *Neurosci. Lett.* 434, 170-174 (2008).
